# The Matron's Department

**Published:** 1905-10-14

**Authors:** 


### THE MATRON'S DEPARTMENT.

### IX. THE NURSES' HOME.

In planning all new hospitals and in reconstructing old ones it is now the invariable custom to separate the nurses' quarters entirely from the main hospital building. The advance of asepticism has brought with it the logical demand that the same scrupulous cleanliness shall be exacted in the nurses' home as in the hospital wards, and the desirability of attracting and retaining good nurses has added privacy and comfort to their quarters.

A good nurses' home is provided with its own staff of servants, and with kitchen premises in which all meals in the home are prepared. It contains a refectory or dining-hall, common rooms for sisters and nurses respectively, separate sitting-rooms for the senior sisters, and separate bedrooms furnished as bed-sitting-rooms for each member of the staff. The rooms of those on night duty are on the top floor or in a wing shut off from the rest of the house. A sick-room, conveniently situated on the first floor, is available for any who are ill.

It is clear that a home of this description, indisputably the best for its purpose, which is that of housing a contented and healthy staff, entails upon the matron the responsibility of two distinct households. She cannot be in two places at once, and it is essential in these large establishments that some one in authority should be continually on the spot if all is to work smoothly. The matron will therefore be obliged to delegate some of her duties to a home sister or assistant housekeeper, whose duties may be combined or not, according to the size of the institution.

The duties of the home sister comprise:—

The supervision of all the rooms in the nurses' home, together with that of the domestic staff and charwomen.

Conducting preparatory classes for probationers, correcting papers set, and looking through note-books.

The care of any who are ill.

Carving and presiding at meals.

Distribution of letters at frequent intervals from letter-box.

Charge of linen and bedding in nurses' home; direction of needlewoman.

To this may be added such assistance as may be required with the matron's correspondence.

The following is atypical day of a home sister in a London hospital:—

7.40 a.m.—Sisters' breakfast.
8 a.m.—On duty. See scrubbers; go round hospital, and nurses' home. See any sick member of nursing or domestic staff.
8.40 a.m.—Matron's office. Distribute letters (letter-box cleared 8.30 a.m., 10.30 a.m., 1 p.m., 3 p.m., 5 p.m., 7 p.m., 9 p.m.). Write orders for repairs, etc. Give out Convalescent Home letters, etc.
9.30 a.m.—Doctor comes if required. The rest of the morning is spent in going round the rooms in the nurses' home. Twice a week class for nurses 10 to 11 a.m.
12.30 p.m.—Nurses' first dinner.
1 p.m.—Nurses' second dinner.

2 p.m.—Matron's office. During the afternoon, correspondence, assist with visitors, etc., etc. Twice a week, lecture at 6 p.m.
7.30 p.m.—Sisters' supper.
8 p.m.—Nurses' first supper.
8.30 p.m.—Nurses' second supper.
9 p.m.—Give out rations for night nurses. Matron's office.
10 p.m.—Lock up down stairs.
10.30 p.m.—Turn out gas in nurses' home and lock up.
*Off duty alternately afternoon and evening.*

It will be seen that illness in the nurses' home makes a great difference in the work of the home sister. A clean bill of health in the household sets her free for many extra duties.

We have quoted this as a specimen of a good home, well organised, under the charge of a capable home sister. But in the present state of hospital development the matron will be exceptionally fortunate if she finds herself in a post where everything is arranged on these satisfactory lines. She is much more likely to be in a position where the comfort of her nurses is a thing to be struggled for and where she has to cope single-handed with the double duties entailed by the hospital and nurses' quarters.

There are compensations in being obliged to overcome difficulties. And the principal compensation which lies in making the best of bad nurses' quarters is that the matron is compelled to exercise her individual gifts, and may, if she is dexterous, and cares enough about it to take a great deal of pains, succeed in producing with the poorest materials just that delicious air of home comfort conspicuously absent in some expensively equipped nurses' homes. She may not be able to isolate altogether the night nurses' quarters, but she may arrange that the work on their floor is finished before they go to bed, that doors in the vicinity are fitted with india-rubber pipings, and that a strip of felt shall deaden any unavoidable footfalls. She may provide their windows with suntight blinds or curtains, and take some pains to see that their meals are appetisingly served, light, and easy of digestion. There is little doubt that one of the main causes of sleeplessness in nurses on night duty is indigestion, caused by the derangement of their ordinary meal-times. Hence any hard or heavy kind of food, such as salt beef or suet pudding, should be omitted from their dietary. The cubicle system has manifold disadvantages for nurses. But the matron may find herself compelled to make the best of it. Much can be done by daily supervision, by tact in placing those together who are congenial companions, and by firm discipline. There is little use in putting printed papers of rules against the wall. The spirit of mutual courtesy must reign, and it is for the matron to instil this by example as well as by taking notice in private of any breach of consideration for others which may occur. Where the cubicle system is in force the bedroom cannot be treated as a bed-sitting-room and the arrangement of the common sitting-room becomes a matter of great importance. We have seen countless nurses' sitting-rooms all over the country ranging from the very elegant to the distinctly shabby, but we have never seen one which came up to our idea of comfort. The essential feature of a sitting-room as a place to sit in appears to be lost sight of in planning these big apartments. They are for the most part reception-rooms where clusters of people may lean back in lounge chairs or receive an occasional visitor. But the nurse who has an hour to spare before going out has probably many little things to do, a letter to write, or notes to prepare for her lecturer, or a bit of sewing. She must fetch everything she wants, probably from a long distance, and then may often find no spare seat where a good light is available, for the sin of lighting such rooms by a chandelier from the top is too prevalent. The nurses' sitting-room, if it is to take the place of a private room, should have a separate low desk or tiny table with locked drawer, assigned to each occupant. These tables could be grouped in sets of two or four with a low-hung gas or electric bracket to each set round the room and provided with stuffed cane armchairs sufficiently easy to give rest to the back without entailing the ridiculous attitudes which make all employment impossible. Think of the comfort to a nervous woman of knowing on entering a crowded room that her own corner is waiting for her, with books and work ready of access. It is the sense that the place is her own which makes the essence of home, and the matron who can produce this feeling among her nurses will be well repaid for her trouble by increased gentleness and contentment among her staff.

A newspaper club is an important feature of the common room, and among even a small staff a halfpenny a week each will produce wonders in the way of variety. If for no other reason but that of widening the range of conversation the matron should not allow this institution to drop through. It is impossible to deal fully with the question of recreation, so different are the possibilities with regard to locality. Cycling is perhaps the ideal recreation as it refreshes the mind, invigorates the whole system, and rests tired feet more effectually than any other form of exercise. By a little contrivance on the part of those in authority room could generally be found for the bicycles of the staff and the success of the cycling club at Guy's, proves that no disadvantages as regards situation need prove insuperable. Room can often be found for an asphalt tennis court where no grass is available and this is an excellent resource all the year round. In some hospitals, notably the Hospital for Paralysis, Maida Vale, and the Great Northern Central Hospital, roof gardens have proved a great boon. Whatever form of recreation may be available the matron may and should do much to smooth away difficulties, and without interfering with the liberty of her nurses when they are off-duty must keep a watchful eye lest any abuse creeps in. Her object is to keep the staff thoroughly healthy in body and mind, and whatever can help to that end is part of her business as head of the family.

The days when nurses were "not expected to be ill" have passed away and every matron must now train herself to recognise those beginnings of illness which are easily checked if taken in hand at once. The system in force at King's College Hospital, which grants the first-year probationer a late morning in bed from time to time, and whenever fatigue declares itself, is worthy of imitation. A

day in bed is understood to be better than drugs after any long-continued strain and often saves time in the end. Doubtful cases should be reported without delay to the doctor, and should the doctor himself observe symptoms of illness in any nurse which have escaped the matron, it should be well received.

It is the duty of the matron to keep under strict observation any nurses who are engaged with typhoid cases, and by perpetual admonition and inspection to enforce precautionary measures. It can hardly be doubted that the numerous instances which come to light of typhoid infection communicated to nurses are due to want of vigour in the superintendence.

